# Prediction of *Streptococcus uberis* clinical mastitis treatment success in dairy herds by means of mass spectrometry and machine-learning

**DOI:** 10.1038/s41598-021-87300-0

**Published:** 2021-04-08

**Authors:** Alexandre Maciel-Guerra, Necati Esener, Katharina Giebel, Daniel Lea, Martin J. Green, Andrew J. Bradley, Tania Dottorini

**Affiliations:** 1 School of Computer Science, University of Nottingham, Jubilee Campus, Wollaton Rd, Nottingham, NG8 1BB Nottinghamshire UK; 2 School of Veterinary Medicine and Science, University of Nottingham, College Road, Sutton Bonington, LE12 5RD Leicestershire UK; 3 Quality Milk Management Services Ltd, Cedar Barn, Easton Hill, Easton, BA5 1DU Wells UK; 4 Digital Research Service, University of Nottingham, College Road, Sutton Bonington, LE12 5RD Leicestershire UK

**Keywords:** Proteomic analysis, Computational models, Data mining, Machine learning, Protein analysis

## Abstract

*Streptococcus uberis* is one of the leading pathogens causing mastitis worldwide. Identification of *S. uberis* strains that fail to respond to treatment with antibiotics is essential for better decision making and treatment selection. We demonstrate that the combination of supervised machine learning and matrix-assisted laser desorption ionization/time of flight (MALDI-TOF) mass spectrometry can discriminate strains of *S. uberis* causing clinical mastitis that are likely to be responsive or unresponsive to treatment. Diagnostics prediction systems trained on 90 individuals from 26 different farms achieved up to 86.2% and 71.5% in terms of accuracy and Cohen’s kappa. The performance was further increased by adding metadata (parity, somatic cell count of previous lactation and count of positive mastitis cases) to encoded MALDI-TOF spectra, which increased accuracy and Cohen’s kappa to 92.2% and 84.1% respectively. A computational framework integrating protein–protein networks and structural protein information to the machine learning results unveiled the molecular determinants underlying the responsive and unresponsive phenotypes.

## Introduction

Mastitis is the most prevalent endemic disease in the dairy industry impacting productivity, profitability and cow welfare. A small group of bacteria (*Staphylococcus aureus*, *Streptococcus uberis, Streptococcus dysgalactiae, and Escherichia coli*) are responsible for 80% of mastitis cases^1^, among these *S. uberis* has been consistently found as the major pathogen in dairy farms around the world^2,3^.

Infections may go from subclinical to severe and life-threatening. The causes underlying the progression of the disease are not yet known, however, is likely to be determined by the pathogen (i.e. species, strain, virulence etc.) and host factors (i.e. immune status)^4,5^. Besides, the aetiology of clinical mastitis varies with time, within and between countries and seasons^6,7^. Such variation poses several challenges such as necessitating researchers to continue reshaping of the recommended mastitis control programme over time and importantly the need for a reliable diagnostic technique. The lack of fast, affordable and effective detection of the pathogen at the time of clinical presentation leaves the veterinarian in the condition to primarily rely on educated guesses. Amongst the most serious consequences of this is the indiscriminate administration of broad-spectrum antibiotics, which result in increased antimicrobial resistance. Mastitis is the most common reason for antimicrobial resistance drug therapy for cows on US dairy farms^8^.

Moreover, very little is known about the factors affecting the likely outcome of treatment for clinical mastitis cases. Although differences in cure rates depend on the different drugs used, the key factors found to be relevant in determining the outcome of treatment of clinical cases are the time when treatment is initiated and the duration of treatment, with more rapid initiation of treatment and longer treatment duration, both being associated with improved cure rates^9,10^. In contrast to clinical mastitis, a better understanding of the factors influencing the likely outcome of the treatment has been achieved for subclinical mastitis. Where, the type of pathogen, the duration of infection and treatment, the timing of treatment, the number of affected quarters, the parity, somatic cell count (SCC), showed all to be correlated to the likely outcome treatment^11–13^.

The SCC can be used as a coarse estimator of presence/absence of mastitis infection because SCC increases when defence cells migrate from the blood to the mammary glands to combat the disease^14^. However, other factors such as the stage of lactation, age, breed, parity, stress, season, milk transportation and management, and diurnal variations can also increase the SCC levels in milk^15–17^. The literature indicates that, although SCC can be used as a coarse estimator of presence/absence of mastitis infection, it is hard to use SCC to infer gravity of infection on a continuous scale. Moreover, it has not been possible so far to identify a relationship between SCC and the risk of transmission of the infection^18^. However, the diagnostic power of SCC may be increased by combining it with other information. For example, Ebrahimi et al.^19^ used machine learning (ML) on SCC and other data gathered at the time of milking (i.e. the amount of protein, fat and lactose, electrical conductivity), to determine whether a cow had subclinical mastitis.

Although several factors influencing the outcomes of treatment have been identified, fast, affordable and effective diagnostic solutions for day-to-day analysis to discriminate between responsive and unresponsive strains of *S. uberis* and strains failing to respond to treatment are still missing. A recently proposed solution for diagnosis of bovine mastitis is matrix-assisted laser desorption/ionisation time-of-flight (MALDI-TOF) mass spectrometry. MALDI-TOF has been proven effective in the diagnosis of clinical mastitis in cows and has been also shown capable of performing at strain level^18,20,21^.

ML techniques could improve diagnosis including in the case of bovine clinical mastitis since these methods can learn from data patterns and exploit the information embedded in the peaks retrieved from MALDI-TOF data^22^.

However, to the best of our knowledge, no study in the literature uses ML techniques to differentiate responsive from unresponsive strains of *S. uberis* from cows with clinical mastitis.

This work illustrates a novel diagnostic solution for the dairy cow, targeting the discrimination between infections caused by responsive and unresponsive strains of *S. uberis*, where responsive means that the strain is likely to respond positively to intramammary antibiotic treatment, whilst unresponsive identifies the strains which are likely to fail to respond. The solution is powered by supervised ML and is designed to work using as input a combination of MALDI-TOF spectra and additional background data related to the cows under examination.

The diagnostic solution has been developed using data collected from a sample of 90 individuals as part of a previous research project^23^. The data includes MALDI-TOF spectra, SCC values and additional background information related to the individual cows. We first showed that, although individual SCC values were known to be only partially reliable indicators of infection, aggregations of SCC values acquired at multiple time points during the treatment could be used to build a statistical model that reliably discriminates between responsive and unresponsive cases. We then used this finding to generate responsive/unresponsive labels for the isolates to be able to use in supervised learning. Then we trained a variety of ML models using MALDI-TOF spectra of labelled isolates and compared the diagnostics performance of the resulting classifiers by using cross-validation. We first showcased the performance of 9 traditional ML methods using the spectral features obtained by the intensity of the statistical different peaks in terms of the Welch’s *t*-test. We then demonstrated that the performance of all classifiers can be further improved by i) implementing a new way to encode MALDI-TOF spectra as inputs to the ML models, and ii) adding further background data collected from the individual cows, also as inputs to the classification model. Specifically, improvement in diagnostics performance could be achieved by adding information about parity, SCC prior to treatment (i.e. SCC in the most recent milk sample before the MALDI-TOF analysis), and the number of positive clinical mastitis history. Finally, we showed that the peaks of the MALDI-TOF spectra which were singled out by the optimized ML models as the most relevant for discrimination between responsive and unresponsive strains, actually corresponded to bacteriocin and ribosomal proteins, suggesting that immunity and drug resistance features can be correlated to the different phenotypes, and indicating that the information found relevant by the ML model had also biological meaning.

## Results

### Data source

In this study, data from clinical mastitis cases on 26 farms located in England and Wales were used (see Methods). Within each farm, the number of cows with *S. uberis* associated clinical mastitis ranged from one to nineteen. A final dataset set of 90 cows with *S. uberis* associated mastitis and their corresponding bacterial isolates was considered to develop the models illustrated in this work. There were 38 *S. uberis* isolates that were classed as responsive and 52 isolates that were classed as unresponsive, categorised according to the 3 SCC values following the clinical mastitis case (see Methods). The available data for each *S. uberis* associated clinical mastitis event included MALDI-TOF spectra, SCC values from milk recording data collected at multiple time points and a series of additional background information related to the history of each individual cow (i.e. parity, SCC of the previous lactation, and count of positive mastitis history).

### Generation of features used as input for the machine learning

The original MALDI-TOF raw spectra were obtained using Bruker technology. Peak lists with paired mass/charge (m/z) ratios and corresponding intensity values were extracted from the raw spectra as specified in the Methods Section.

To discriminate between infections caused by responsive and unresponsive strains of *S. uberis*, we selected as input data for the ML approach a combination of MALDI-TOF spectra and additional background data related to the cows under examination.

Two MALDI-TOF spectral feature selection schemes (Fig. S-1) were considered to select spectral input data to discriminate between responsive and unresponsive *S. uberis* isolates: (1) the intensity of the peaks found to be statistically different between the two classes as per Welch ‘s *t*-test and Wilcoxon test and, (2) from the list of statistical different peaks, an m/z binning scheme was used to create further spectral features. In addition, a third feature selection approach by adding further background data collected from the individual cows were also used as inputs to the classification model.

### Identification of statistically significant peaks (input spectral features)

The Welch’s *t*-test and Wilcoxon test analysis produced a list of 10 statistically significant peaks. Table 1 shows, for each one of the 10 statistical significant peaks, the average and standard deviation for each class (responsive and unresponsive), the overall proportion of appearance (due to a signal to noise threshold each point is considered a peak if its intensity is higher than 10% of the maximum intensity) in the data set, the proportion of appearance in each class and the *p*-value of the statistical tests (Welch’s *t*-test and Wilcoxon test).

**Table 1:** Peak statistic report.

Mass (kDa)	PTTA	PWKW	Ave1	Ave2	StdDev1	StdDev2	PA	PA1	PA2
3.9487	2.25E-10	2.07E-09	10.61	8.79	0.90	1.36	46.67%	78.95%	23.08%
4.7406	5.73E-10	1.61E-08	29.38	20.56	6.48	5.49	98.89%	100.00%	98.08%
5.3775	0.008235	0.005979	13.16	14.82	2.70	3.00	92.22%	89.47%	94.23%
5.9547	0.000021	2.73E-05	16.95	19.27	2.27	2.52	98.89%	97.37%	100%
6.3860	0.011298	0.000872	11.62	12.64	1.29	2.15	91.11%	92.11%	90.38%
6.7178	0.000028	5.54E-05	15.82	17.96	1.77	2.56	100%	100%	100%
6.8411	0.00244	0.000898	14.58	16.31	2.18	2.87	96.67%	94.74%	98.08%
7.9054	0.046388	0.074268	13.01	12.02	1.58	2.70	83.33%	94.74%	75.00%
8.1396	0.0000897	6.59E-05	14.72	16.60	1.50	2.51	98.89%	97.37%	100%
9.4878	0.000123	7.06E-05	27.14	20.97	6.75	7.48	96.67%	100%	94.23%

*PTTA* is the *p*-value of Welch ‘s *t*-test; *PKWK* is the *p*-value of Wilcoxon test; *index 1* refers to class responsive; *index 2* refers to class unresponsive; *Ave* is the overall intensity average; *Ave1* is the intensity average of class responsive; *Ave2* is the intensity average of class unresponsive; *StdDev* is the overall intensity standard deviation; *StdDev1* is the intensity standard deviation of class responsive; *StdDev2* is the intensity standard deviation of class unresponsive; *PA* is the overall proportion of appearance; *PA1* is the proportion of appearance of class responsive; *PA2* is the proportion of appearance of class unresponsive.

### Identification of spectral features using a binning scheme (input spectral features)

We used an m/z binning scheme (see Methods) to select the spectral features from the list of the 10 statistical different peaks (Table 1). A total of 10 bins were created:

- 3 bins did not carry any peaks (2–3 kDa, 10–11 kDa and 11–12 kDa) because the intensity level was consistently below 10% of the maximum intensity
- 2 bins carried multiple peaks (5–6 kDa and 6–7 kDa)
- 5 bins had a single peak (3–4 kDa, 4–5 kDa, 7–8 kDa, 8–9 kDa and 9–10 kDa)

**Figure 1:**
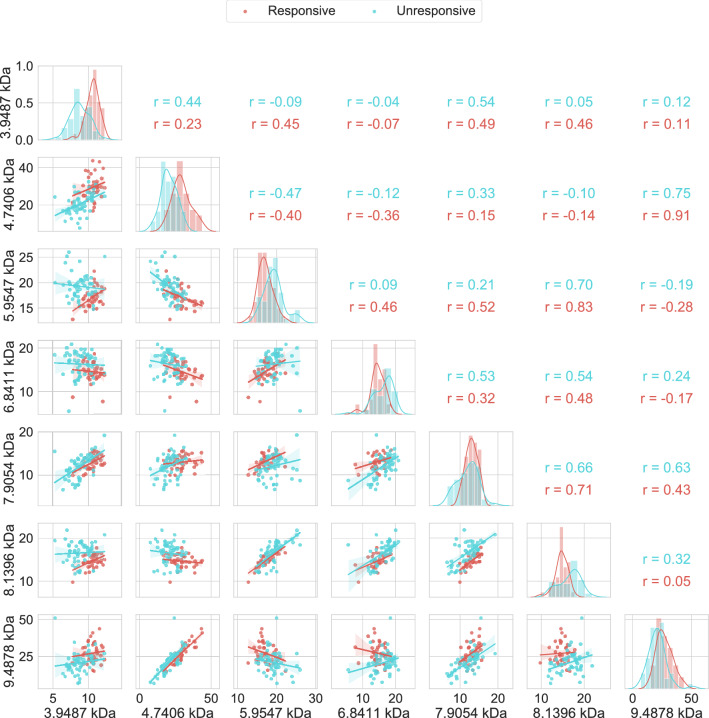
Pair plot pairwise relationship between the most common peaks in the binning method. The responsive and unresponsive intensities are coloured red and blue, respectively. The plots in the lower triangle show the relationship between two peaks for responsive and unresponsive classes; this relationship is in terms of the normalized intensity. The diagonal shows the distribution of the normalized intensity of each peak; the y-axis indicates the frequency while the x-axis indicates the normalized intensity. The upper triangle shows the person correlation for the relationship between two peaks for responsive and unresponsive classes.

Therefore, we could only use 7 bins containing peaks to create spectral features data. Table 2 shows the proportion of appearance of the peaks in the two bins containing multiple peaks. Figure 1 shows the seven peaks that most appear in the bins using a pair plot graph, the plot shows each peak paired with all the other peaks, with the diagonal containing the distribution of each peak. From this pairwise comparison it is possible to observe that 3 peaks (3.9487 kDa, 4.706 kDa and 9.4878 kDa) have the intensities of the responsive samples higher than those of the unresponsive samples, while for the other four peaks (5.9547 kDa, 6.8411 kDa, 7.9054 kDa and 8.1396 kDa) the intensities of the unresponsive samples are the same or higher than those of the responsive ones. Moreover, peaks 4.706 kDa and 9.4878 kDa have the highest positive Pearson correlation for both responsive (r = 0.91) and unresponsive (r = 0.75).

**Table 2:** The proportion of appearance (PA) of each peak in the spectral features data for the two bins which contained multiple peaks.

Bin	5–6 kDa		6–7 kDa		
Mass (kDa)	5.3775	5.9547	6.3860	6.7178	6.8411
PA	7.78%	92.22%	1.11%	12.22%	86.67%

### Identification of external features, background data related to the cows under examination, (input external features)

We used three external features: (1) parity, (2) SCC of the prior to the clinical diagnosis but in the same lactation, and (3) count of previous clinical mastitis cases; to evaluate whether they could lead to an increase in performance when used together with MALDI-TOF peaks acquired from the pipeline described on Fig. S-1. The three sets of features were categorised according to specific metrics as detailed in the methods and are shown in Table 3. The prediction performance of each classifier was evaluated considering accuracy, sensitivity, specificity and Cohen’s kappa score using the proposed external features and the results are shown in Supplementary Fig. 2.

**Table 3:** The median and standard deviation values of the external feature metric for both classes.

	Parity	SCC of the prior to the clinical diagnostic	Clinical mastitis cases
Responsive	3 ± 1*.*35	3 ± 1*.*25	1 ± 0
Unresponsive	4 ± 1*.*67	3 ± 2*.*09	2 ± 1*.*49

**Figure 2:**
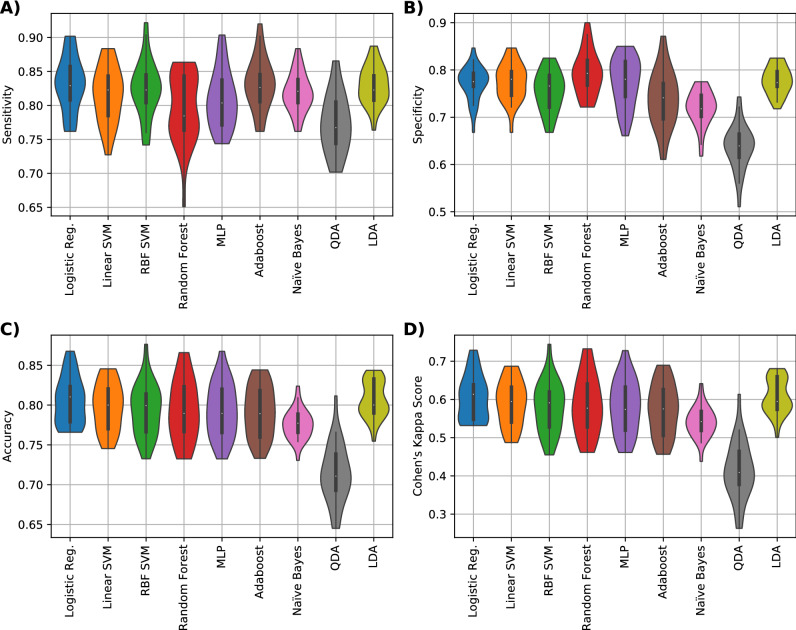
Performance metrics results for the data set composed with only all statistical relevant peaks. (**A**) Sensitivity, (**B**) Specificity, (**C**) Accuracy and (**D**) Cohen’s kappa values of nine different classifiers (logistic regression, linear SVM, RBF SVM, random forest, MLP NN, Adaboost, QDA and LDA) are shown.

### Discrimination between responsive and unresponsive *S. uberis* isolates using a solution powered by supervised machine learning

To develop the classifiers, we trained a variety of ML models using MALDI-TOF spectra of labelled isolates and compared the diagnostics performance of the resulting classifiers by using cross-validation. The median, maximum and minimum values of the performance metrics of each classifier were calculated and are shown for each violin plots. The prediction performance of each classifier was evaluated considering accuracy, sensitivity, specificity and Cohen’s kappa score. We first showcased the performance of 9 traditional ML methods using the spectral features obtained by the intensity of the statistical different peaks in terms of the Welch’s *t*-test. On the 10 peaks scheme, the best classifier in terms of the Cohen’s kappa score was logistic regression that achieved 61.35%, this classifier had an accuracy of 81.03%, sensitivity of 82.91% and specificity of 77.5% (Fig. 2).

**Figure 3:**
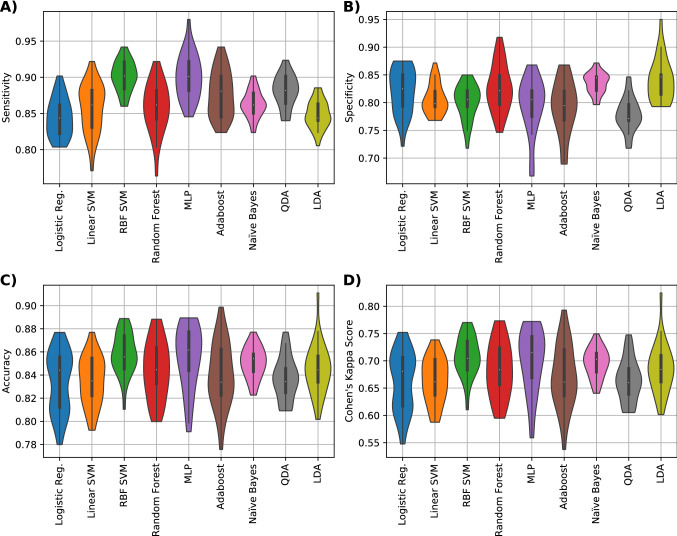
Performance metrics results for the data set composed with the features acquires from the binning scheme. (**A**) Sensitivity, (**B**) Specificity, (**C**) Accuracy and (**D**) Cohen’s kappa values of nine different classifiers (logistic regression, linear SVM, RBF SVM, random forest, MLP NN, Adaboost, QDA and LDA) are shown.

**Figure 4:**
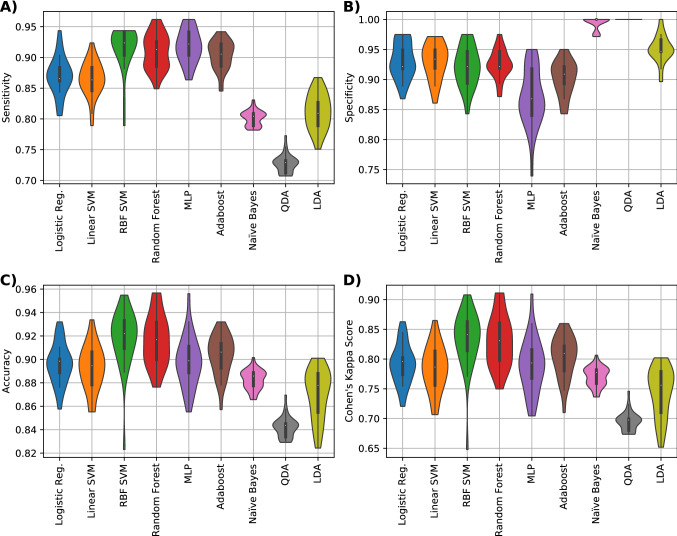
Performance metrics results for the data set composed with the binning features and the three external features. (**A**) Sensitivity, (**B**) Specificity, (**C**) Accuracy and (**D**) Cohen’s kappa values of nine different classifiers (logistic regression, linear SVM, RBF SVM, random forest, MLP NN, Adaboost, QDA and LDA) are shown.

We then demonstrated that the performance of the classification could be further improved by using the newly implemented binning method to encode MALDI-TOF spectra as inputs to the ML models. Specifically, for the binning scheme, the best classifier in terms of the Cohen’s kappa score was the MLP that achieved 71.5%, this classifier had an accuracy of 86.2%, sensitivity of 90.1% and specificity of 80.5% (Fig. 3). Finally, an additional improvement in diagnostics performance could be achieved by adding further background information about parity, SCC of previous lactation (i.e. SCC in the most recent milk sample before the MALDI-TOF analysis), and the number of positive mastitis history, to the binning method. Namely, the addition of parity, SCC of prior to the clinical diagnostic and count of positive clinical mastitis history, improved the classification performance to 84.1% in terms of the Cohen’s kappa score (an increase of 12.6%), an accuracy of 92.2% (an increase of 6%), sensitivity of 92.4% (2.3% increase) and specificity of 92.1% (an increase of 11.6%) with a Support Vector Machine classifier with Radial Basis Function kernel (RBF SVM) (Fig. 4).

To estimate the competitiveness and efficiency of the classifiers we have computed the model prediction run time (Supplementary Table 1). Most predictive models work fast and often completed their calculations in real time showing high efficiency of the classifiers. LDA and QDA showed best running performances but have lower classification performances than the other learners. Notably, the RBF-SVM learner that showed to have higher classification performance than the other learners, also showed to have competitive running time (18.1048 s).

### Identification of the proteins found to correspond to the MALDI-TOF spectral peaks recognised as discriminant by the trained classifiers

The ten peaks identified as providing optimal discrimination between two classes were further analysed to identify their cognate *S. uberis* proteins. Both MALDI-TOF spectral feature methods share the same 10 statistical different peaks, with the difference being that on the binning method 7 peaks at a time were used (either 5.3775 kDa (7.78% times) or 5.9547 kDa (92.22% times) were used for 5-6 kDa bin, and either 6.3860 kDa (1.11% times), 6.7178 kDa (12.22% times) or 6.8411 kDa (86.67% times) were used for 6-7 kDa bin) in the individual isolate due to maximum peak selection method for each bin. When compared to the reference *S. uberis 0140 J* proteome, three peaks (3.9487 kDa, 4.741 kDa and 5.9547 kDa) could not be matched to any protein as they did not meet the criteria described in the Methods section.

**Figure 5:**
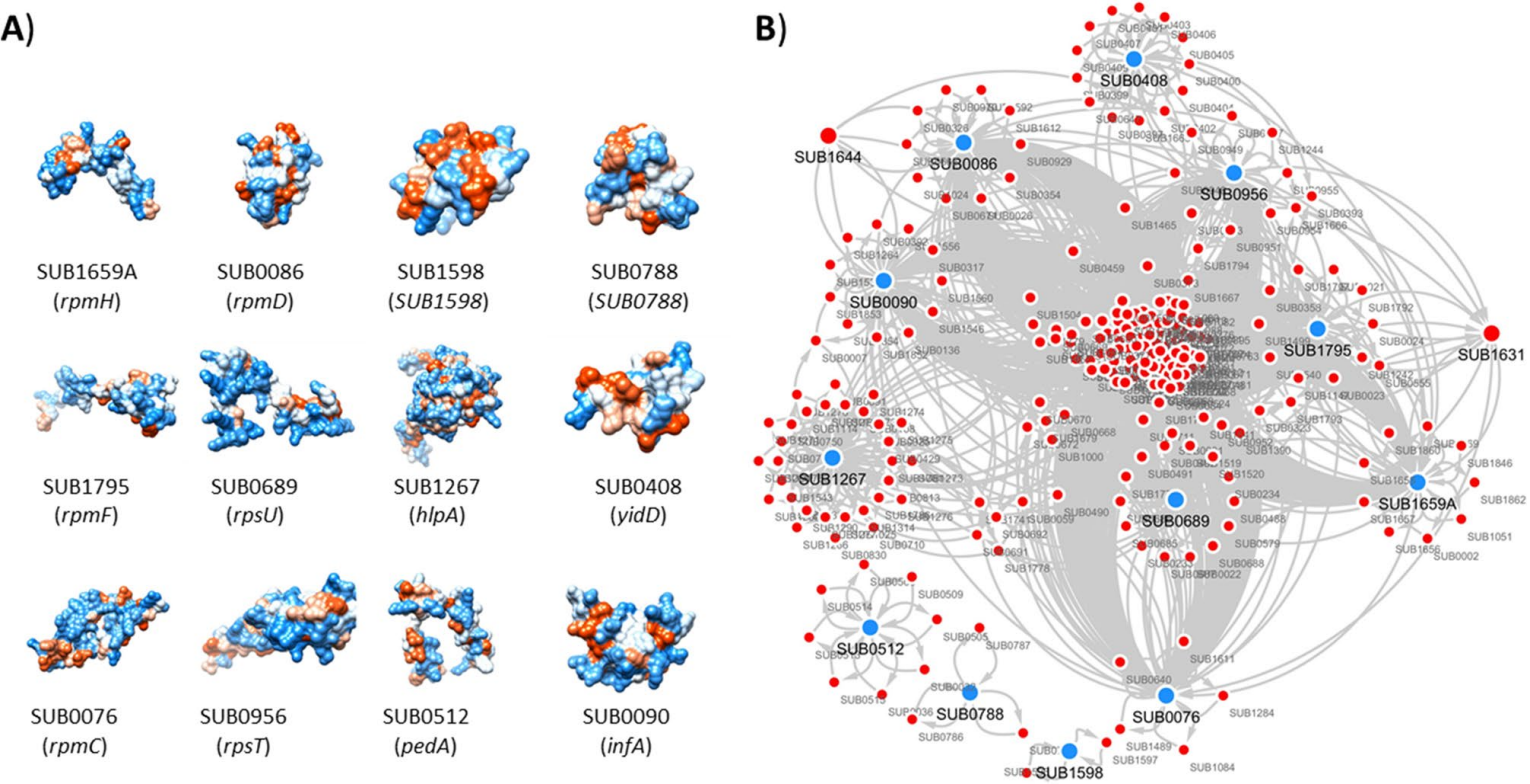
Protein–protein interaction network of the discriminant proteins found to correspond to the MALDI-TOF spectral peaks recognized as significant by the trained classifiers. (**A**) 3D homology structures of the 12 discriminant proteins. (**B**) The Protein–protein interaction network showing the 12 discriminant proteins (blue circles) and the 239 *S. uberis* proteins (red circles) to which they interact with (first shell interacting partners). Amongst these first shell interacting partners, two proteins—SUB1644 (ksgA) and SUB1631 (fusA)—are related to antimicrobial resistance.

The remaining 7 out of 10 peak masses could be matched to 12 different proteins (Table 4). Specifically, peak 6386.04 Da could be matched to three proteins (SUB0086, SUB1598 and SUB0788), peak 8139.57 Da could be matched to three proteins (SUB0956, SUB0512 and SUB0090) and peak 9487.76 Da could be matched to two proteins (SUB1267 and SUB0408). While peaks 5377.47 Da, 6717.79 Da, 6841.15 Da and 7905.41 Da matched to one protein in *S. uberis 0140 J* proteome. The entire procedure is described in the Methods section. Peak masses, their corresponding proteins with gene ID and locations predicted by PSORTB v3.0 are described in Table 4. Two out of these twelve proteins were hypothetical proteins (SUB1598 and SUB0788) while ten proteins had known functions: four of them were 50S ribosomal proteins (SUB1659A, SUB0086, SUB1795 and SUB0076) two of them were 30S ribosomal proteins (SUB0689 and SUB0956), and the four remaining proteins were bacteriocin (SUB0512), translation initiation factor (SUB0090), DNA-binding (SUB1267), and a membrane protein with insertion efficiency factor (SUB0408). The predicted three-dimensional models of these proteins are shown in Fig. 5A.

**Table 4:** MALDI-TOF peak masses and their cognate *S. uberis* proteins.

Peak mass	Protein (MW)	Locus ID (Gene ID)	Location
5377.47 Da	50S ribosomal L34 (5377.39 Da)	SUB1659A (*rpmH*)	Cytoplasmic
6386.04Da^a^	50S ribosomal L30 (6385.58 Da)	SUB0086 (*rpmD*)	Cytoplasmic
6386.04Da^a^	Hypothetical Protein (6381.39 Da)	SUB1598 (*SUB1598*)	Unknown
6386.04Da^a^	Hypothetical Protein (6382.44 Da)	SUB0788 (*SUB0788*)	Cytoplasmic
6717.79 Da	50S ribosomal L32 (6717.67 Da)	SUB1795 *(rpmF*)	Cytoplasmic
6841.15 Da	30S ribosomal S21 (6840.95 Da)	SUB0689 (*rpsU*)	Cytoplasmic
7905.41 Da	50S ribosomal L29 (7904.23 Da)	SUB0076 (*rpmC*)	Cytoplasmic
8139.57Da^a^	30S ribosomal S20 (8138.30 Da)	SUB0956 (*rpsT*)	Cytoplasmic
8139.57Da^a^	Bacteriocin (8145.49 Da)	SUB0512 (*pedA*)	Extracellular
8139.57Da^a^	Translation initiation factor IF-1 (8141.46 Da)	SUB0090 (*infA*)	Cytoplasmic
9487.76Da^a^	DNA-binding HU (9485.94 Da)	SUB1267 (*hlpA*)	Cytoplasmic
9487.76Da^a^	Membrane protein (9490.26 Da)	SUB0408 (*yidD*)	Cytoplasmic

^a^Peaks having more than one protein matches based on criteria of 0.2% molecular weight distance range from the peak mass.

### The protein–protein interaction (PPI) network of the proteins found to correspond to the MALDI-TOF spectral peaks recognized as significant by the trained classifiers

To gain basic knowledge of the pathogenic and molecular function of the proteins corresponding to the MALDI-TOF spectral peaks recognized as significant by the trained classifiers, we generated the PPI network (Fig. 5B). A total of 239 *S. uberis* proteins have been found to interact with the 12 discriminant proteins (first shell interacting partners). The analysis of the PPI network showed that most of the proteins found to correspond to the significant MALDI-TOF spectral peaks SUB0076 (*rpmC*), SUB0086 (*rpmD*), SUB0090 (*infA*), SUB0408 (*yidD*), SUB0689 *(rpsU*), SUB0956 (*rpsT*), SUB1267 (*hlpA*), SUB1659A (*rpmH*) and SUB1795 (*rpmF*) interact directly with each other (Fig. 5B). Only 3 out of 12 proteins (SUB0512, SUB0788 and SUB1598), which are bacteriocin and hypothetical proteins did not interact with any other discriminant proteins.

The analysis of the connectivity of this network (251 proteins) showed slightly lower connectivity (clustering coefficient 0.409) than the complete *S. uberis* PPI network (clustering coefficient 0.438). The clustering coefficient provides a scale of interconnectivity network (between 0 and 1, where 1 means all neighbours are connected and 0 means no connection of the neighbours at all)^24^. The average number of neighbours per protein was 6.048 in the network containing the discriminant proteins and 30.142 in the complete *S. uberis* PPI network. In terms of network density, the values ranged between 0.024 (network containing the discriminant proteins) and 0.017 (complete *S. uberis* PPI network) and for the network heterogeneity the values ranged between 2.724 (network containing the discriminant proteins) and 1.321 (complete *S. uberis* PPI network).

**Figure 6:**
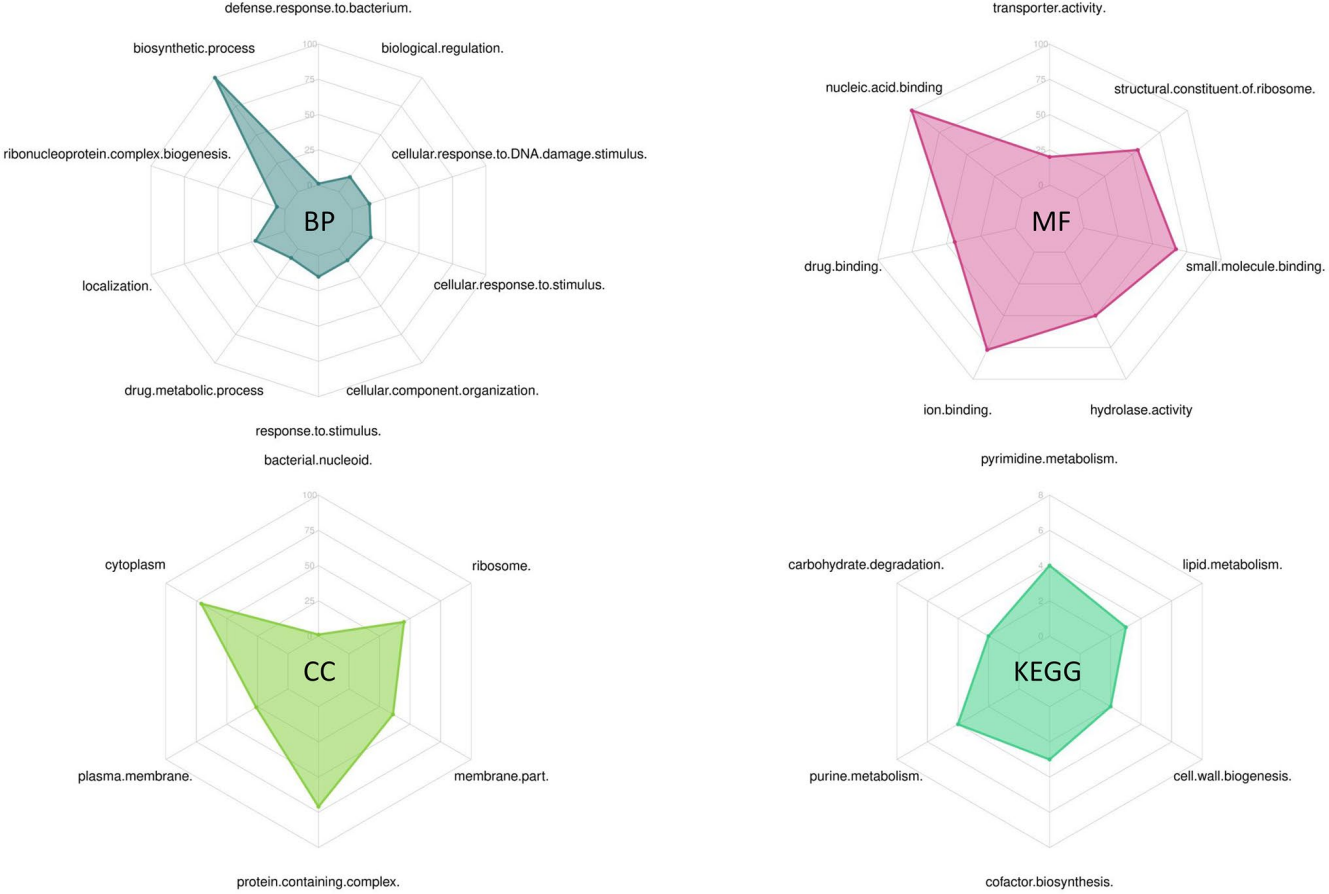
Gene Ontology (GO) analysis including the cellular component (CC), molecular function (MF), and biological process (BP) and KEGG pathway enrichment analysis of the genes encoding the 251 proteins present in the PPI. In each ontology, the enriched categories and the number of genes populating them are shown. Likewise, the enriched KEGG pathways and the number of genes in them are indicated. The GO was created using FMSB library (version 0.7.0—https://cran.r-project.org/web/packages/fmsb/index.html) in R^75^.

The presence of such network connectivity may suggest that the discriminant proteins are not just co-expressed but are also involved in functional complexes and pathways. To address this Gene Ontology (GO)^25^ analysis including the cellular component (CC), molecular function (MF), and biological process (BP) and Kyoto Encyclopedia of Genes and Genomes (KEGG)^26^ pathway enrichment analysis were carried out for the genes encoding the 251 proteins present in the PPI (see Fig. 6). The results showed enrichment in the molecular function of binding (drug, ion and nucleic acid), biosynthetic process, response to stimulus and drug metabolic process. Moreover, purine-pyrimidine metabolism, cofactor biosynthesis, lipid metabolism, carbohydrate degradation and cell wall biogenesis were found as significantly enriched KEGG pathways (Fig. 6).

Finally, in order to interpret the molecular mechanisms underlying the drug resistance phenotypes, we investigated if the *S. uberis* proteins interacting with the discriminant proteins are involved in antibiotic resistance. Interestingly, homologs of SUB1631 (elongation factor G—*fusA*) was previously found to be related to fusidic acid^27^ resistance by preventing drugs from binding their target^28^ whereas homologs of SUB1644 (dimethyladenosine transferase—*ksgA*) is thought to confer resistance to aminoglycoside antibiotics kanamycin^29^ and kasugamycin^30^. SUB1644 (*ksgA*) and SUB1631 (*fusA*), were predicted as common first neighbour proteins of SUB0956 (*rpsT*), SUB0086 (*rpmD*), SUB0090 (*infA*) and SUB0689 (*rpsU*). In addition, SUB1631 (*fusA*) was predicted as first neighbour of SUB0076 (*rpmC*), SUB1659A (*rpmH*) and SUB1795 (*rpmF*).

## Discussion

*S. uberis* strains failing to respond to antibiotic treatment and able to progress to a persistent infection are increasingly frequent in dairy farms. Therefore, identification of *S. uberis* strains that fail to respond to the treatment with broad-spectrum antibiotics is essential to guide clinical management. The continuous accumulation of omics data with associated metadata in public repositories requires a reference computational platform to fastly integrate and meta-analyse this type of data. We have developed the first iteration of a computational platform to meet this challenge. By combining ML and MALDI-TOF mass spectrometry we showed that spectral profiles acquired from clinical isolates can be used for the successful recognition of *S. uberis* strains that fail to respond to conventional treatment and those that are clinically responsive. We showed that aggregations of SCC values acquired at multiple time points could be used to generate “responsive” and “unresponsive” labels for the isolates to be able to use in supervised learning. Our computational solution adopts novel spectral feature schemes based on using the spectral together with metadata collected in the field, combined with the discriminatory power of a new binning scheme to achieve high prediction results when compared with 10 statistical significant peaks obtained using conventional statistical peaks selection methods. Classifiers developed on using the binning scheme achieved higher classification performance in terms of accuracy, sensitivity, specificity and Cohen’s kappa score when compared with classifiers based on spectral features with 10 statistical different peaks. For the first time, we demonstrated that external features, such as parity, SCC of previous lactation and number of clinical mastitis cases can increase the overall performance of the classifiers if used together with the spectral features obtained by the MALDI-TOF. The results for this analysis showed that RBF SVM was the best classifier in terms of Cohen’s kappa score of 84.1% (with an increase of 12.6% with respect to the binning performance), an accuracy of 92.2% (an increase of 6% with respect to the binning performance), sensitivity of 92.4 (with a 2.3% increase) and specificity of 92.1% (with an increase of 11.6%). The increased performance is possibly explained by recent findings^16^, showing that milk SCC can be influenced by parity, lactation stage, health and other factors, regardless of whether the cow is infected or not^16,17^. Nevertheless, our method indicates that, for this data set, external features such as parity, number of mastitis cases and SCC before the clinical diagnostic, could possibly be related to the infection, since the prediction performance increased. Likewise, compared to multiparous cows, young cows produce less milk and have a lower milk SCC^16,17^. Also, SCC can increase depending on the stage of lactation, which has been linked with a cow’s immune response in preparation for calving to enhance the mammary gland defence mechanism^17^.

Furthermore, using our platform we identified the molecular factors affecting the likely outcome of treatment for clinical mastitis cases and uncovered additional drug-resistant signatures within the PPI network.

This demonstrates the platform’s ability to generate hypotheses that may not only support better decision making and treatment selection but also may expand our knowledge of the molecular basis of progression to a persistent infection.

The proteins found to be discriminant between responsive and unresponsive S. *uberis* isolates (i.e. SUB1659A (*rpmH*), SUB0090 (*infA*), SUB0086 (*rpmD*) and SUB0956 (*rpsT*)) and the resistome interactions (SUB1631 (*ksgA)* and SUB1644 (*fusA*)) revealed are new but in many cases involve known genes characterised for their AMR function in other species.

Interestingly, the homologs of the discriminant proteins were previously found involved in antibiotic resistance. Specifically, *rpmH* was found to be involved in the regulation of polyamine biosynthesis^31^, which is associated with increased antimicrobial resistance in *E. coli* by blocking channels in the outer membrane^32^. *RpmC* was previously shown to bind puromycin, an aminonucleoside antibiotic, in both *E. coli* and *Bacillus stearothermophilus*^33^. Also, *rpmF* was previously shown to be involved in membrane lipid synthesis^34^ and persister cell formation which may help bacteria to survive antibiotic treatment^35^. *RpmF* depleted *E. coli* mutants were shown to increase susceptibility against ampicillin, gentamicin, levofloxacin, metronidazole, rifampicin and sulfamethoxazole in different studies^35–37^. *RpsU* was shown to be related to motility and biofilm formation in *Bacillus subtilis*^38^, stress resistance in *Listeria monocytogenes*^39^, metronidazole resistance in *Helicobacter pylori*^40^, and daptomycin and vancomycin susceptibility in *Staphylococcus aureus*^41,42^*.* Finally, the homologue of the DNA-binding HU protein was shown to be correlated with beta-lactam resistant *E. coli* strains^43^.

Although the first neighbour interactions of SUB1644 (*ksgA*) and SUB1631 (*fusA*) with our discriminant *S. uberi*s proteins have not been experimentally validated yet, homologs of SUB1644 (*ksgA*) and SUB0090 *(infA*) in *E. coli* K12 W3110 was found to be interacting^44^. Likewise, SUB0090 (*infA*), SUB0956 (*rpsT*), SUB0086 (*rpmD*) and SUB1659A (*rpmC*) were shown to be interacting with SUB1631 (*fusA*) in *E. coli* K12 W3110 and *S. cerevisiae*^44,45^.

However, this is the first time these molecular determinants have been identified as possibly being involved in the progression to a persistent *S. uberis* infection. Interestingly, the proteins *rpmH*, *rpmC*, *rpmF* and *rpsU* were previously suggested as potential biomarkers to discriminated clinically relevant *Campylobacter coli* clades based on sample sources such as farm animals, humans or environmental water^46^. The role of these proteins as potential biomarkers to discriminate different strains is two-fold: provide an incentive for additional detailed experimental analysis and inform a new line of the experimental campaign.

While our computational solution successfully discriminates responsive and unresponsive *S. uberis* isolates, there are limitations that need to be approached as future work. Firstly, we used cows from England and Wales and thus it is unknown how the performance will be when analysing cows from other countries with different antibiotic usage. A wider repertoire of observations will help to understand the robustness of the method and its potential clinical applicability. Secondly, while our study provided a basis for hypothesis testing, a wider m/z spectra range to investigate high molecular weight determinants (beyond 20 kDa) will provide a means to dissect all the possible proteins involved in sustaining the responsive and unresponsive phenotypes and offer an even more performant diagnostic classifier. Finally, our approach relies on the protein information present in public repositories and thus does not provide the investigation beyond repositories annotated entries.

Genomics, transcriptomics, proteomics and metadata coupled with machine learning, are advancing and their integration can offer invaluable information well beyond of what can be assessed individually with the distinct approaches. For example, previous studies showed the significant associations among phenotypic AMR profiles of *S. uberis*, genomic features and epidemiological data such as SSC, sample type (e.g. lactation, post-mastitis and post-calving)^47,48^. Analogously, Perry JA, Westman EL and Wright GD^49^ showed that gene expression level is a key determinant of phenotypic resistance. Although, *S. uberis 0140J* displays a lower number of mobile genetic elements when compared with other pyogenic streptococci^50^, integrative and conjugative elements (ICEs) and their association with macrolide, lincosamide, and tetracycline resistance were observed^51^. Several studies have shown that resistant genes or pathogenicity islands can have different GC content and that these genomic features can potentially be used to track transmission/acquisition of the AMR determinants^52^.

When developing a predictive tool is becoming highly important to consider how we could benefit from the integration of different omics approaches and metadata to develop a solution that not only predicts the AMR phenotypes with high-accuracy but also accounts for the dynamic nature of AMR.

Overall, the results of this study meet the critical need to integrate various kind of omics data with associated metadata to provide a robust, effective and rapid means to discriminate responsive and unresponsive strains of *S. uberis*. The developed computational solution utilizes a unique combination of MALDI-TOF omics data, ML and background metadata, structural analysis, functional enrichment analysis and PPI analysis. This type of data integration and data analysis will become increasingly more needed in the near future.

Our computational platform may open up new strategies for the development of fast and effective day-to-day data processing and diagnostic solutions to study other human and animal pathogens.

## Methods

### Data collection: MALDI-TOF data set

For this work, clinical mastitis data collected from 52 dairy farms in England and Wales between 2004 and 2005 available from a previous study^23^ was used. Clinical mastitis cases associated with *S. uberis* where selected and the *S. uberi*s isolates corresponding to the clinical mastitis cases in the datasets were used to generate the MALDI-TOF spectra.

An initial search of the data revealed 180 isolates with mastitis caused by *S. uberis.* All 180 isolates were allocated an SCC score using the SCC count of three-monthly milk recording events following the identified case of *S. uberis* mastitis. In order to take into account antibiotic treatment taking effect, if the first SCC was > 200 k but the milk recording was taken within 14 days of the clinical mastitis case, then this recording would be ignored. The 4^th^ SCC was then used instead as long as the recording had taken place within 120 days of the clinical mastitis case. The following process was applied to allocate an SCC score to each case of *S. uberis* mastitis (n = 180):

- Initially, we gave a score which reflects the different SCC (number of cells/ml) recorded after a case a of clinical mastitis 0 − 100 k → 1
- 100 − 200 k → 2
- 200 − 500 k → 3
- 500 − 1000 k → 4
- or > 1000 k → 5

The scores given for each of the three SCCs post case were then added up and divided by the maximum sum possible (15), resulting in a final SCC score between 0.2 and 1 for each case of *S. uberis* associated mastitis. For the purpose of this study, isolates with a score between 0.2 and 0.3 were allocated into the “responsive” group (n = 38, mean score = 0*.*22 ± 0*.*03) and isolates with a score between 0.4 and 1 were allocated in the unresponsive class (n = 52, mean score = 0*.*83 ± 0*.*2). Based on these scores, cases of mastitis which had a high level of confidence of responsive and unresponsive were identified. This final dataset of 90 isolates were then recovered and the mass spectrometry was generated. In a small number of cases in the unresponsive category, the isolate from the case from which the score had been generated could not be recovered – in this case an isolate from a recurrent case in that quarter was used (though that case may have resulted in a lower SCC score). These 90 isolates with clinical mastitis cases caused by *S. uberis* was used to develop the models illustrated in this work. This comprised of data from 26 farms.

*The S. uberis* isolates corresponding to the 90 *S. uberis* mastitis cases identified were retrieved from storage (− 80 °C) on microbeads and cultured by a commercial milk laboratory. Selected *S. uberis* were incubated on blood agar at 37 °C for 18 to 24 h. Protein extraction was conducted as previously described^53^. The pure cultures were then analysed using the Time-of-flight (TOF) MALDI mass spectrometer (Bruker Daltonics, Billerica, MA), Microflex – Flex Control Version 3.4, Bruker Daltonics. For each *S. uberis* isolate, 6 technical replicate profiles were generated from 240 desorption’s per replicate (6 × 40 shots). Spectra were compared visually using Biotyper 3.1 (Bruker Daltonics). Those with insufficient resolution, low intensity, or substantial background noise were removed. Technical replicates were further compared using composite correlation indices (CCI) to remove dissimilar spectra with CCI < 0.99^54^. The samples had at least remaining 3 out of 6 technical replicates were included in the analysis.

### Data pre-processing

Mass spectrometry pre-processing step was performed by using MATLAB and Bioinformatics Toolbox Release 2017b, The MathWorks, Inc., Natick, Massachusetts, United States. The analysis was done for 90 samples (38 responsive *S. uberis* and 52 unresponsive *S. uberis*) with each sample having 3 to 6 replicates.

Initially, each replicate was treated individually (351 spectra in total) and after normalisation, we computed the mean value of each group of replicates to have the original 90 samples of the data set, as suggested by^55^. To account for any issues with the spectra quality a visual assessment was performed on each individual spectrum before the pre-processing steps. Our pre-processing method is based on the following 8 steps:

1. M/Z Cropping: the mass range was cropped to be between 2 and 12 kDa.
2. Resampling: the data was upsampled from 13,740 points to 20,000 points.
3. Baseline Correction: for each biological isolate, baseline correction was applied by using a window of 200 Da with a step size of 200 Da to shift the window. The quantile method (10% value) was used to find the likely baseline value in every window. Shape-preserving piecewise cubic interpolation approximation was applied to regress the varying baseline. The regressed baseline was not smoothed. The resulting baseline was subtracted from the spectrum.
4. Normalization: the area under the curve (AUC) of every spectrum was normalized to the median and post-rescaled such that the maximum intensity was 100.
5. Mean Computing: the replicates of each biological isolate were averaged.
6. Noise reduction: each sample was denoised using least-squares polynomial with a window of 35 Da and a 2-degree polynomial function.
7. Alignment: to align the spectrograms, a set of reference peaks was required. The peaks occurring in at least 30% of the spectra were used as a reference. The alignment was estimated using the default values of msalign function in the Bioinformatics Toolbox. This alignment step is important to overcome miscalibration issues of the mass spectrometry that can lead to variations in the relationship between the observed M/Z value and the true time-of-flight of the ions
8. Peak Detection: to retain a reasonable intensity a signal-to-noise ratio threshold was defined at 10% to discard all peaks below it. Therefore, since the spectra were previously normalized to an overall maximum intensity of 100, any point below 10 is considered noise. A minimum distance of 20 Da between neighboring peaks was set, i.e., two peaks must be at least 20 Da apart to be considered different.

### Spectral features

After detecting all the peaks in each spectrum, a peak list report was prepared similarly to ClinProTools 3.0^56^. The *p*-value of the *t*-test for each peak was calculated. The Welch’s *t*-test null hypothesis states that the means of two normally distributed populations (in this case the two classes, responsive and unresponsive, for each peak) are equal but have unknown variances. The alternative hypothesis is that the data from the two classes come from populations with unequal means. A 0.05 threshold was chosen for statistical significance. Peaks significantly different between the responsive and unresponsive classes were identified from the raw spectra data, using the Welch’s *t*-test with a *p* < 0*.*05. The *p*-value and the *t*-test of the paired mass/charge (m/z) ratio and corresponding intensity values for all the peaks in each one of the 90 spectra have been calculated. Specifically, the peaks were selected if present in at least 30% of all spectra, as suggested by ClinProTools 3.0. The selected peaks were further preprocessed to have zero mean and unit variance. Such peaks represented the spectral features used in the classification analysis.

### Binning

The peaks were used in two different ways. In the first, all the peaks whose difference was recognized as statistically relevant were used. In the second, an m/z binning scheme to select peaks was used. The spectra were binned using a bin size of 1 kDa from the range of 2–12 kDa so that 10 bins were created. The maximum intensity value of the peaks within each bin was used as a spectral feature. For both schemes, the spectral features were further pre-processed to have zero mean and unit variance before the classification process.

### External features: cow metadata collection

The three external features related to the cows background were collected in the field and are: (1) parity, (2) SCC of the prior to the clinical diagnosis but in the same lactation, and (3) count of previous clinical mastitis cases. The three sets of features were categorised according to the following metrics:

- Parity: 1, 2, 3, 4 or 5 + 
- Clinical Mastitis cases: 1, 2, 3, 4 or 5 + 
- SCC of prior to the clinical diagnostic: No recording → 0; 0 − 50* k → *1; 50 − 100* k → *2; 100 − 200* k → *3; 200 − 400* k → *4 or > 400* k → *5

### Classification methods

The performance of the classifiers, naïve Bayes^57^, linear and non-linear (RBF kernel) support vector machines (SVM)^58^, random forest^59^, multi-layer perceptron neural networks (MLP)^60^, AdaBoost (AdaBoost-SAMME version)^61^, logistic regression^62^, linear discriminant analysis (LDA)^63^ and quadratic discriminant analysis (QDA)^63^, was investigated using the scikit-learn library in Python^64^.

For the classifiers, the following set of values were employed for the hyper-parameter searches:

- Logistic Regression: inverse of regularization strength C = [0.001, 0.01, 0.1, 1, 10, 100, 1000].
- Linear SVM: penalty parameter of the hinge loss error C = [0.001, 0.01, 0.1, 1, 10, 100, 1000].
- Random Forest and Adaboost: Number of estimators = [2, 4, 8, 16, 32, 64].
- MLP Neural Network: α (L2 penalty parameter) = [0.001, 0.01, 0.1, 1, 10, 100], learning rate (initial learning rate used to control the step size in updating the weights with adam solver) = [0.001, 0.01, 0.1, 1] and hidden layer sizes = [10, 20, 40, 100, 200, 300, 400, 500].
- Non-linear SVM with RBF kernel: γ (RBF kernel coefficient) = [0.0001, 0.001, 0.01, 0.1] and C (L2 penalty parameter) = [0.001, 0.01, 0.1, 1, 10, 100, 1000].
- Naive Bayes, LDA and QDA: do not have hyper-parameters.

### Prediction performance

The prediction performance of each classifier was evaluated by considering the following indicators, assuming P and N as the total number of positive (responsive) and negative (unresponsive) isolates, respectively and using T for true (correct) and F for false (wrong) predictions:

- Sensitivity (True Positive Rate) = TP / P
- Specificity (True Negative Rate) = TN / N
- Accuracy = (TP + TN)/(P + N)
- Kappa = (*p*_*o*_ – *p*_*e*_)/(1-*p*_*e*_) where *p*_*o*_ = (TP + TN)/(P + N) and p_*e*_ = (P*(TP + FN) + N*(FP + TN)) /(P + N)^2^

### Performance analysis

Nested Cross-validation (NCV)^65^ was employed to assess the performance and select the hyper-parameters of the proposed classifiers. In NCV, there was an outer loop split of the data set into test and training sets. For each training set, a grid search (inner loop) was run, to find the best hyper-parameters of the classifier using accuracy as a performance metric. Then, the test set was used to score the best classifier found in the inner loop.

Thirty iterations were carried out, wherein each iteration an NCV was employed. The inner loop of the NCV found the best hyper-parameters of each classifier (when suited) using stratified threefold cross-validation (66% for training and 33% for validation); the outer loop measured the accuracy, sensitivity, specificity and kappa using fivefold stratified cross-validation (20% for testing and 80% for training), to compare all the classifiers^66^.

### Identification of the proteins found to correspond to the MALDI-TOF spectral peaks recognised as discriminant by the trained classifiers

A dedicated bioinformatics pipeline was developed to find correspondences between individual peaks selected by the ML-based classifiers and actual proteins of *S. uberis*. First, amino acid sequences of the proteins in the *S. uberis* 0140 J proteome, which was considered the model bovine mastitis strain^67^, were retrieved from the PATRIC database. The molecular weights of the proteins were calculated using the Compute pI/Mw tool on ExPASy^68^. The proteins were filtered in the range of ± 200 Da of the mass of individual peaks. Then, N-terminal methionine cleavage was controlled using the online prediction tool TermiNator^69^ and the theoretical molecular weights of the proteins were re-calculated using compute pI/Mw tool according to the presence or absence of the initial methionine. Finally, proteins with a maximum of 0.2% difference in mass to the individual peaks for the successful identification of correspondence were selected.

To further investigate the function of the identified proteins, we studied protein–protein interactions (PPI) as previously described^20^. The PPI dataset of *S. uberis* 0140J was obtained from the STRING database^70^ and nodes (proteins) with interaction scores lower than medium confidence level (interaction scores < 0.400) were filtered out. The remaining nodes (proteins) were analysed in Cytoscape 3.7.1 based on the following parameters: the average number of neighbours, clustering coefficient, network density and network heterogeneity^71^. The average number of neighbours represented the mean connectivity value of a protein in the network. The clustering coefficient provided a scale of interconnectivity in the network (between 0 and 1, where 1 meant all neighbours connected and 0 meant no connection of neighbours at all)^72^. Network density was the normalized version of the average number of neighbours, and proteins with no connectivity were rated 0, while proteins with lots of connections were given 1. Network heterogeneity indicated the variance of connectivity and the more hub nodes, the greater value it had^73^.

The antimicrobial related nodes in the PPI network were characterized by blasting against the AMR genes in the antibiotic classes (such as aminoglycosides, tetracycline, fusidic acid etc.) that were available in ResFinder database^74^. The functions of the genes in the network were annotated with Gene Ontology (GO) terms (biological process, molecular function and cellular component) and KEGG pathways. The GO map was built using the FMSB library (version 0.7.0—https://cran.r-project.org/web/packages/fmsb/index.html) in R^75^. Finally, to gain a more in-depth understanding of the protein functions, 3D homology models for the discriminant proteins were built by using the Swiss-Model repository^76^. The 3D Models of all discriminant proteins were visualized and edited in UCSF Chimera^77^. PSORTb v3.0 was used to predict cellular locations of the discriminant proteins^78^.

## Supplementary Information

Supplementary Information 1.

Supplementary Information 2.

Supplementary Information 3.

## Data Availability

The data underlying the results presented in the study are available from Quality Milk Management Services Ltd (QMMS) - email: enquiries@qmms.co.uk.
