# Supplementary material for: Prediction of Streptococcus uberis clinical mastitis treatment success in dairy herds by means of mass spectrometry and machine-learning
Source: Sci Rep. 2021 Apr 8;11:7736. doi: 10.1038/s41598-021-87300-0 (PMC8032699; doi:10.1038/s41598-021-87300-0)
Supplement: Supplementary file 1 — Supplementary Information 1. [file 41598_2021_87300_MOESM1_ESM.pdf]

Prediction of *Streptococcus uberis* clinical mastitis treatment success in dairy herds by means of mass spectrometry and machine-learning

Alexandre Maciel-Guerra[1§], Necati Esener[2§], Katharina Giebel[3], Daniel Lea[4], Martin J. Green[2], Andrew J. Bradley[2,3] and Tania Dottorini[2*]

[1]University of Nottingham School of Computer Science, Jubilee Campus, Wollaton Rd, Nottingham, Nottinghamshire NG8 1BB, UK

[2]University of Nottingham School of Veterinary Medicine and Science, College Road, Sutton Bonington, Leicestershire, LE12 5RD, UK

[3]Quality Milk Management Services Ltd, Cedar Barn, Easton Hill, Easton, Wells, BA5 1DU, UK

[4]Digital Research Service, University of Nottingham, College Road, Sutton Bonington, Leicestershire,LE12 5RD, UK

§ co-first authors

[*]corresponding author (email: tania.dottorini@nottingham.ac.uk)

Supplementary Table S1. Prediction run time of the different classifiers. The prediction run time for each classifier was calculated over 30 iterations and using the different sets of features to appreciate the differences in time performance. Time is measured in seconds. The run time of the classifiers was calculated on Processor: Intel(R) Xeon(R) CPU E5-1620 v4 @ 3.5GHz; RAM: 32Gb; Windows 10.

Classifier	Prediction time /s (spectral features - peaks)	Prediction time /s (spectral features - binning)	Prediction time /s (spectral - binning - and external features)
Logistic Regression	23.09925	18.89913	23.53048
Linear SVM	6.060186	5.332506	5.168754
RBF SVM	19.34117	17.05391	18.1048
Random Forest	110.6596	96.37867	96.55977
MLP	14466.1	14889.09	11925
AdaBoost	89.82297	94.38135	92.17564
Naïve Bayes	1.324485	0.746325	0.804546
QDA	1.147809	0.786384	0.868869
LDA	1.021395	0.775956	0.60504
