# Supplementary material for: Prediction of Streptococcus uberis clinical mastitis treatment success in dairy herds by means of mass spectrometry and machine-learning
Source: Sci Rep. 2021 Apr 8;11:7736. doi: 10.1038/s41598-021-87300-0 (PMC8032699; doi:10.1038/s41598-021-87300-0)
Supplement: Supplementary file 2 — Supplementary Information 2. [file 41598_2021_87300_MOESM2_ESM.pdf]

Prediction of *Streptococcus uberis* clinical mastitis treatment success in dairy herds by means of mass spectrometry and machine-learning

Alexandre Maciel-Guerra[1§], Necati Esener[2§], Katharina Giebel[3], Daniel Lea[4], Martin J. Green[2], Andrew J. Bradley[2,3] and Tania Dottorini[2*]

[1]University of Nottingham School of Computer Science, Jubilee Campus, Wollaton Rd, Nottingham, Nottinghamshire NG8 1BB, UK

[2]University of Nottingham School of Veterinary Medicine and Science, College Road, Sutton Bonington, Leicestershire, LE12 5RD, UK

[3]Quality Milk Management Services Ltd, Cedar Barn, Easton Hill, Easton, Wells, BA5 1DU, UK

[4]Digital Research Service, University of Nottingham, College Road, Sutton Bonington, Leicestershire,LE12 5RD, UK

§ co-first authors

[*]corresponding author (email: tania.dottorini@nottingham.ac.uk)

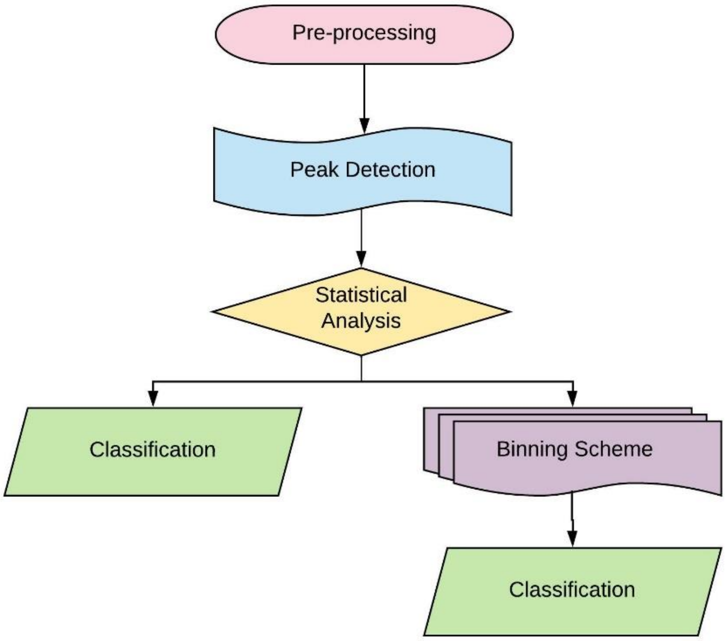

Figure S-1. Pipeline describing the steps for MALDI-TOF spectra analysis used in this study, with the two spectral features methods used for data analysis. (Created in Lucidchart, www.lucidchart.com)
