# Supplementary material for: Prediction of Streptococcus uberis clinical mastitis treatment success in dairy herds by means of mass spectrometry and machine-learning
Source: Sci Rep. 2021 Apr 8;11:7736. doi: 10.1038/s41598-021-87300-0 (PMC8032699; doi:10.1038/s41598-021-87300-0)
Supplement: Supplementary file 3 — Supplementary Information 3. [file 41598_2021_87300_MOESM3_ESM.pdf]

Prediction of *Streptococcus uberis* clinical mastitis treatment success in dairy herds by means of mass spectrometry and machine-learning

Alexandre Maciel-Guerra[1§], Necati Esener[2§], Katharina Giebel[3], Daniel Lea[4], Martin J. Green[2], Andrew J. Bradley[2,3] and Tania Dottorini[2*]

[1]University of Nottingham School of Computer Science, Jubilee Campus, Wollaton Rd, Nottingham, Nottinghamshire NG8 1BB, UK

[2]University of Nottingham School of Veterinary Medicine and Science, College Road, Sutton Bonington, Leicestershire, LE12 5RD, UK

[3]Quality Milk Management Services Ltd, Cedar Barn, Easton Hill, Easton, Wells, BA5 1DU, UK

[4]Digital Research Service, University of Nottingham, College Road, Sutton Bonington, Leicestershire,LE12 5RD, UK

§ co-first authors

[*]corresponding author (email: tania.dottorini@nottingham.ac.uk)

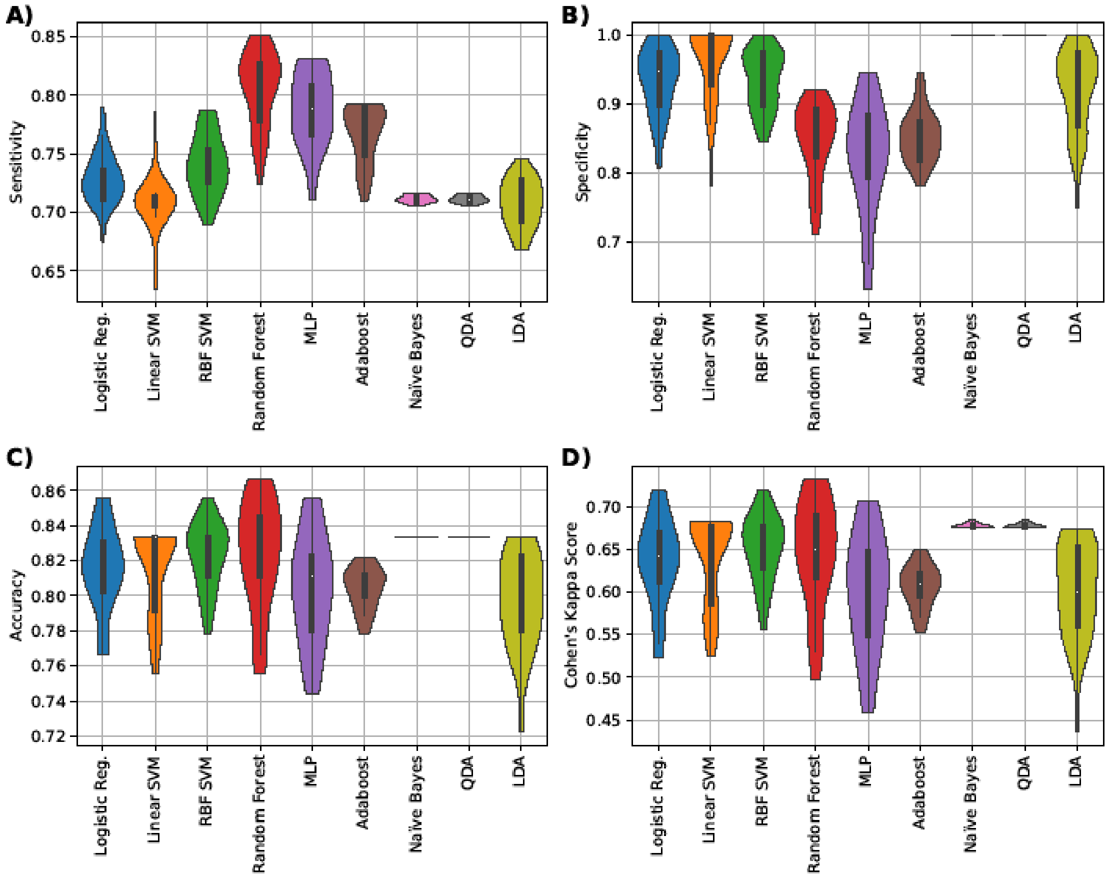

Figure S-2. Performance metrics results for the data set composed with the three external features. A) Sensitivity, B) Specificity, C) Accuracy and D) Cohen's kappa values of nine different classifiers (logistic regression, linear SVM, RBF SVM, random forest, MLP NN, Adaboost, QDA and LDA) are shown.
